# Novel Nutrition Education Approaches for Health Promotion: From Investigating Problems to Finding Solutions

**DOI:** 10.3390/nu13124423

**Published:** 2021-12-10

**Authors:** George Moschonis, Emmanuella Magriplis, Antonis Zampelas

**Affiliations:** 1 Department of Dietetics, Nutrition and Sport, School of Allied Health, Human Services and Sport, La Trobe University, Melbourne, VIC 3086, Australia; G.Moschonis@latrobe.edu.au; 2 Laboratory of Dietetics and Quality of Life, Department of Food Science and Human Nutrition, Agricultural University of Athens, 11588 Athens, Greece; emagriplis@aua.gr

The aim of the *Nutrients* Special Issue “Implications of Nutrition Education, for Health, Behavior, and Lifestyle” is to publish original research articles and reviews that report the design and implementation of nutrition education intervention programs and their effectiveness in terms of lifestyle, health, and wellbeing. The importance of exploring this field in depth is highlighted in this Special Issue, since nutrition education represents the main means for training individuals and groups on the principles of good nutrition based on their needs, thus making nutrition information digestible and usable in everyday life.

According to the World Health Organization, nutrition is defined as “the intake of food in relation to the body’s dietary needs”. An adequate, well-balanced diet is a cornerstone of good health, has a great impact on wellbeing, and is reflected by dietary intake and behavior. Diet and behavior are highly modifiable factors that can help to prevent the emergence of new cases of lifestyle-related chronic conditions or ameliorate their progression. These factors can be modified using population-specific nutrition education techniques.

Currently, a wide discrepancy exists in nutrition-related advice that originates from a wide range of different sources, many of which are unreliable and scientifically invalid, further underlining the need for nutrition education that is tailored to the specific requirements of the target population. Furthermore, although health professionals play a critical role in educating individuals in the clinic, the community, or a healthcare facility, nutrition education is not fully integrated with the training programs of most healthcare professions. This partly explains the public’s considerable confusion on what is considered correct nutritional advice, which is exacerbated by these discrepancies.

Considering these challenges, fourteen very interesting papers have been published in this Special Issue, addressing different aspects of Nutrition Education, using various methodological approaches and study designs. More specifically, the Special Issue includes six observational studies, reporting various aspects or determinants that affect the development and implementation of nutrition education initiatives. In one of these studies, Carolina Archundia Herrera M. et al. [1] conducted one-on-one, semi-structured, open-ended, in-depth interviews, as part of which participants expressed their views and preferences on the content (i.e., related to nutrition, physical activity, mental health, foot care, and consequences of type 2 diabetes (T2D)), features (i.e., in terms of understanding the context, explicit information, individualized, hands-on learning, and whether these are applicable, realistic, incremental, and practical), and other components (i.e., access to a multidisciplinary team, setting goals, monitoring and tracking of progress and be held accountable, one-on-one sessions, group support, maintenance/follow-up) of effective future diabetes management nutrition education programs, as well as on appropriate policy change required to support these initiatives.

Furthermore, Lyndsey D. Ruiz et al. [2] has developed and pilot-tested a food literacy curriculum for high-school-aged adolescents. The curriculum that was entitled “*Teens CAN: Comprehensive Food Literacy in Cooking, Agriculture, and Nutrition*” contained 12 modules of experiential lessons and application activities within three topics/lessons, i.e., agriculture, focusing on the food supply chain and food environments; nutrition, focusing on food groups, as well as on nutrients of concern for underconsumption; and cooking, focusing on food safety, budgeting, and preparation. The researchers pilot tested the curriculum and reported that it can provide a comprehensive and necessary approach to advancing food literacy in adolescents.

The influence of physical education (PE) classes on students’ healthy dietary habits was investigated in the cross-sectional study by María-Jesús Lirola et al. [3], which was conducted with 3415 students (13–19 years old) attending secondary schools in Spain. The study collected data on the psychological constructs derived from the theory of self-determination and the theory of planned behavior, such as students’ satisfaction and frustration of basic psychological needs, motivation in participating in PE classes, as well as their social cognition and intention of having a healthy diet (e.g., the Mediterranean Diet). The findings of this study showed that a high level of satisfaction perceived in PE classes helped students reinforce their intention of having a healthy diet and therefore generating a perdurable commitment to this habit.

In another cross-sectional study, Ice Yolanda Puri et al. [4] assessed the nutritional practices for the management of people with type 2 diabetes (T2D) by nutrition educators (i.e., *n* = 50 nutritionists) working at the Public Health Clinic (PHC) in Padang, Indonesia. This study showed that one-third of Indonesian nutritionists were delivering daily counseling sessions. Approximately half of the nutritionists conducted a monthly follow-up session for the patients in the previous three months, with each nutritionist educating an average of five to ten people with T2D. The most common nutrition education topics delivered included appropriate menus, as well as the etiology and symptoms of T2D. Almost all nutritionists used leaflets, and about one-third used information posters as tools and materials to facilitate the delivery of nutrition education to the target group of diabetics. Around 71% of counseling sessions lasted 30 min and two-thirds of the sessions included nutrition education. Furthermore, about half of the nutritionists claimed that people with T2D were reluctant to attend individual nutrition education sessions, mainly because they were not interested in the educational tools and materials that were used as part of these sessions. The latter highlights the need for the development of new nutrition and diabetes education tools in Indonesia that will be stimulating for people with T2D to actively participate in the relevant sessions.

In their cross-sectional study, Aleksandra Małachowska and Marzena Jeżewska-Zychowicz [5] tried to investigate whether food experiences in childhood can help understand food choices in adulthood. More specifically, this study examined 443 adults aged 18–65 years, who filled in a modified version of the Comprehensive Feeding Practices Questionnaire (CFPQ) in order to measure adults’ recollections of their feeding experiences during childhood (5–10 years old) and then assess if these experiences are related to food choices in adulthood. Based on the results of this study, “pressure and food reward” and “child control” were associated with a higher intake of sweets and salty snacks in adulthood. On the other hand, “healthy eating guidance”, “monitoring”, and “restrictions” were associated with higher consumption of fresh fruits and vegetables.

Another observational study by Adi-Lukas Kurniawan et al. [6] examined possible associations of nutrition education and its interaction with lifestyle factors on kidney function parameters and cardiovascular risk factors among chronic kidney disease patients. This was also a cross-sectional study conducted with 2176 chronic kidney disease patients at stages 3–5 of the disease. The nutrition education program was implemented by a dietitian in the clinical setting of a hospital in Taiwan. Patients were referred by the case manager to the dietitian in the hospital for one-to-one nutrition education (NE) sessions. They were provided with at least 1 or 2, 30–60 min NE sessions within a year or with extra NE sessions if kidney function parameters were getting worse after follow-up every 3 months. The content of NE at the first counseling session included the evaluation of patients’ diet history and the assessment of their dietary energy, macro-, and micronutrients intake, as well as the assessment of their nutritional status. After the first NE session, an individualized diet plan that focused on the appropriate dietary intake of protein, phosphorus, potassium, sodium, and water was recommended to each patient. Dietary intake was modified and monitored individually and according to the physiological and nutritional status of chronic kidney disease (CKD) patients. Overall, the NE sessions program resulted in an improved estimated glomerular filtration rate in patients who were smoking or were physically inactive. In addition, patients who attended the NE sessions significantly reduced the risk of having high glycated hemoglobin A1c (HbA1c) levels and high concentrations of low-density lipoprotein cholesterol (LDL-C). This study suggested that NE can be an effective strategy that improves kidney function and improves cardiovascular risk in CKD patients.

In addition to observational studies, the Special Issue also includes results from 6 intervention studies. In the first intervention study, Louisa Ming Yan Chung et al. [7] recruited 305 adults aged 19–31 years, who were randomly allocated into two groups. The intervention group attended NE sessions, while their dietary intake was monitored through a mobile application, while the control group received NE sessions delivered as part of usual care. Three hours of NE and 12 weeks of self-dietary monitoring represented the two main activities delivered to the intervention group. The study showed significant decreases in sugar consumption, both in the control and the intervention groups, but the decrease in the experimental group was more pronounced compared to the one observed in the control group. The study also reported significant increases in dietary fiber intake within both groups, but the increase in the intervention group was significantly higher than the increase observed in the control group. A decrease in dietary vitamin C intake was also observed in the control group, while vitamin C and fruit intake increased in the intervention group to a greater extent compared to the changes observed in the control group. Lastly, vegetable intake increased within both groups, but similar to the previous findings, the increase was significantly higher in the intervention compared to the control group. Overall, this intervention study showed that younger adults who receive NE, are more likely to improve their dietary Intake when NE also includes the monitoring of their diet.

In another very interesting intervention study, Fabiola Mabel Del Razo-Olvera et al. [8] investigated the primary barriers of adherence to a structured nutrition intervention in patients with dyslipidemia. The main results of their study indicated that barriers to adhering to the nutritional intervention were (i) lack of time to prepare meals, (ii) eating outside home, (iii) unwillingness to change dietary patterns, and (iv) lack of information about a correct diet for dyslipidaemias. However, all these barriers decreased significantly at the end of the intervention, while the study also showed that following a diet plan which provides at least or more than 1500 kcal per day, is associated with good adherence. In addition, the same study showed that adherence to macronutrients intake varied considerably, with participants expressing difficulty in adhering to the recommended carbohydrate and fat consumption.

The intervention study by Anna Pia Delli Bovi. et al. [9], also published in the Special Issue, used a two-phase quasi-experimental design to examine the efficacy of two 6-month personalized mobile technology protocols in terms of better engagement, adherence to follow-up visits, and improved anthropometric and lifestyle parameters in Italian children, as part of the “PediaFit Pilot Project”. The personalized mobile technology consisted of three personalized/not automated What’s App^®^ (Mountain View, CA, USA) self-monitoring or challenge messages per week. Messages were sent by a dedicated coach and were provided to end-users between three-monthly in-presence regular visits with (PediaFit 1.2) or without (PediaFit 1.1) (Salerno, Italy) monthly free-of-charge short recall visits carried out by a specialized pediatric team. The study enrolled 103 obese children (6–14 years old) recruited in the Pediatric Obesity Clinic and were randomized into an intervention group (IG) (*n* = 24 PediaFit 1.1; *n* = 30 PediaFit 1.2) and a control group (CG) (total *n* = 49). The CG received standard treatment only, which included regular face-to-face visits promoting healthy nutrition and physical activity. The study findings showed that the IG achieved significantly better results than the CG for all study outcomes. Comparison of the two IGs at six months also showed that IG 1.2 had a statistically significantly lower drop-out rate, as well as significantly improved body mass index (BMI), screen time, and fruit and vegetable consumption compared IG1.1. Overall, this intervention study by Anna Pia Delli Bovi. et al. [9] suggested that the combination of messaging through personalized mobile technology with monthly free-of-charge recall visits may improve the prefixed outcomes of mobile technology weight loss intervention programs.

Within the same context, Habiba Ali et al. [10] developed a feasibility study that examined the efficacy of a newly developed technology-mediated lifestyle intervention for overweight and obese young adults in the United Arab Emirates. In this study, 161 University students (18–35 years old) completed a 16-week non-randomized feasibility trial with two arms, i.e., the Rashakaty*-Basic (R-Basic) and the Rashakaty-Enhanced (R-Enhanced) (United Arab Emirates) treatment arm, respectively. Participants in the R-Basic arm received access to a static website that contained educational material on healthier eating and physical activity and questionnaires for completion at the baseline and at the end of the study period. The R-Enhanced intervention was based on social cognitive theory and employed a variety of behavior modification strategies, including self-monitoring, goal setting, self-efficacy, problem-solving, and social support to facilitate changes in diet and physical activity. According to the key findings of this study, there were no differences observed in weight loss between the two arms. However, waist circumference decreased more in the R-Enhanced group than in the R-Basic group. Moreover, changes in knowledge related to sources of nutrients and diet–disease relationships were significantly higher among participants that received the R-Enhanced treatment, whereas R-Enhanced participants also reported more time spent on moderate physical activities, as well as more minutes of walking. Participants in the R-Enhanced group also reported higher scores in social support from friends to reduce fat intake and from family and friends to increase their physical activity levels.

In another randomized community-based controlled trial, Mireia Vilamala-Orra et al. [11] investigated the implementation of the “Stages of Change Model” as part of a NE program delivered to patients with severe mental disorders. The intervention lasted 4 months and consisted of a food education strategy that was aimed in promoting the consumption of fruit and vegetables, as part of 15 group sessions (duration of 90 min per session) that were held on a weekly basis. In this study, it is noticeable that although an increase in motivation towards the intake of fruits and vegetables was observed only in the participants that received the intervention (not in the control group), there were no significant differences observed in the actual intake of fruit, vegetables, or fruits and vegetables combined, which indicates that more research is needed to identify the most appropriate eating intervention to increase the intake of fruit and vegetables in patients with severe mental disorders.

The “4 Your Family” randomized controlled trial by Varagiannis et al. [12] was another intervention study published in the Special Issue, which examined the effects of three different family-based interventions in improving eating behaviors of overweight and obese children. The study included 3 study groups and took place in 3 different settings. Specifically, children in Group 1 received an intervention that was delivered to them by health professionals; children in Group 2 received an intervention in private practice clinics of dietitians, and children in Group 3 received an intervention at home through a website that was specifically developed for this purpose. Children in Group 2, who were received the NE by dietitians in private practice, were most engaged to the program and was also the group achieving the most significant reductions in body weight and waist circumference, assessed by z-score differences at the end of the study compared to baseline. Groups 2 and 3 also achieved a significant reduction in total body fat, assessed using Bioimpedance Analysis, although total energy intake was reduced only in Group 3. This was explained by examining between-group differences in food groups consumed, where Group 2 had achieved most within-group differences. Specifically, children in Group 2 were found to increase consumption of whole wheat cereals and grains, low-fat dairy, while they decreased the consumption of sweets, fast food, and processed meat. Furthermore, children in Group 3 showed increases in their consumption of fruit and low-fat dairy only. Only children in Group 1 had a slight increase in vegetable intake. All intervention groups, however, showed a decrease in total screen time. The authors suggested that more improvements were observed in Group 2.

Lastly, the Special Issue also presents findings from two systematic literature reviews, one of which includes a meta-analysis. The first systematic literature review by Silvia Sánchez-Díaz et al. [13] represents a collection of 14 studies that report on the effects of NE interventions on eating habits, nutrition knowledge, body composition, and physical performance in team sport athletes. Most of the selected studies showed improvements in or maintenance of good eating habits, nutrition knowledge, and body composition in the examined athletes. These findings suggest the implementation of NE interventions in team sport athletes could be an effective strategy to improve their eating habits, nutrition knowledge, and body composition. However, due to the heterogeneity across the included studies regarding sport modality, competition level, age, and sex of athletes, as well as the intervention type adopted (i.e., online or face-to-face), it is difficult to establish optimal NE interventions for each one of the examined outcomes.

The last article by Emilio Abad-Segura et al. [14] was a systematic literature review and meta-analysis, which aimed at summarizing the evolution of scientific production and research trends at a global level, during the last 52 years (1968–2019), on management accounting for a healthy NE. The main finding of this meta-analysis was an exponential trend, especially in the previous decade, with more than 50% of scientific production. Future lines of research had also been identified, namely, (i) investment in health systems, (ii) green label education, (iii) early impact of food insecurity, (iv) WIC (women, infants, and children) nutrition education, (v) food waste audit, and (vii) ecological footprint of food.

In conclusion, despite the barriers and the challenge in adjusting and personalizing NE programs to the needs of the target population, their implementation is of particular importance from a public health perspective. In this regard, NE programs can be tailored and delivered to target groups of different sociodemographic and cultural background and can be proved quite effective in improving dietary habits and, as such, promoting health in both healthy people and patients. New technology can be proved to be an important aspect for the successful and wider implementation of NE programs in the population, but further research is required to identify the most efficient mode of their delivery.
